# Influence of Dexamethasone on Some Reproductive Hormones and Uterine Progesterone Receptor Localization in Pregnant Yankasa Sheep in Semiarid Zones of Nigeria

**DOI:** 10.1155/2017/9514861

**Published:** 2017-10-18

**Authors:** Dauda Yahi, Nicholas Adetayo Ojo, Gideon Dauda Mshelia

**Affiliations:** ^1^Department of Veterinary Physiology and Biochemistry, Faculty of Veterinary Medicine, University of Maiduguri, PMB 1069, Maiduguri, Nigeria; ^2^Department of Theriogenology, Faculty of Veterinary Medicine, University of Maiduguri, PMB 1069, Maiduguri, Nigeria

## Abstract

Dexamethasone is widely used in both veterinary and human medical practices. However, it seems to cause some deleterious effects on pregnancy probably by causing changes in the reproductive hormone levels and their corresponding receptor concentrations. This study investigated the effects of dexamethasone on these parameters. Twenty healthy adult Yankasa sheep comprising 18 ewes and 2 rams were used for this study. Pregnancies were achieved by natural mating after estrus synchronization. Dexamethasone was administered at 0.25 mg/kg body weight on days 1, 3, and 5 during first trimester; days 51, 53, and 55 during second trimester; and days 101, 103, and 105 during the third trimester. Blood samples were collected biweekly for hormonal assay. Uterine biopsies were harvested through caesarean section for immunohistochemical analysis. Results showed that dexamethasone significantly (*p* < 0.05) decreased progesterone concentrations and caused abortion in Yankasa sheep but had no significant (*p* > 0.05) effect on estrogen, while progesterone receptors (PR) were upregulated. The abortion could probably be due to decreased progesterone concentrations as a consequence of the adverse effects on placenta. The PR upregulation may be a compensatory mechanism to increase progesterone sensitivity. It was concluded that dexamethasone should not be used in advanced pregnancy in Yankasa sheep.

## 1. Introduction

Dexamethasone is a fluorinated compound derived from corticosteroid and having 21-carbon steroid skeleton with hydroxyl (OH^−^) or methyl (CH_3_^−^) group attached at C_16_. This compound has virtually no mineralocorticoid effect but remains potent anti-inflammatory and analgesic glucocorticoid with broad significant physiological and therapeutic uses [1, 2]. Therefore, it is used to treat and manage several diseases and medical conditions in both animals and humans [3–5]. Despite the significance of dexamethasone therapies, long applications especially at high doses are associated with some harmful effects in pregnant subjects. The adverse effects range from glucose intolerance to more severe effects like decreased placental weights and efficiency, intrauterine growth restriction (IUGR), and altered hypothalamic-pituitary-adrenal axis [2, 5–11]. The mechanisms underlining some of these deleterious effects during pregnancy are not clear as different species do not always respond to medicines in the same way. However, changes in reproductive hormone levels and their receptor concentrations may be involved. Progesterone and estrogen are chemically classified as steroids and are regarded as the two main reproductive hormones in mammals [12], with progesterone playing a central role in the maintenance of pregnancy. During the course of normal pregnancy, progesterone concentration increases as the pregnancy progresses in order to maintain the integrity of pregnancy by the sustenance of uterine quiescence. However, decreasing progesterone concentration or its receptor (PR) expression and/or activity promotes parturition or abortion [12].

During pregnancy, progesterone is mainly produced by corpora lutea (CL) and placenta and, to a lesser extent, adrenal cortex [13–16]. Estrogen, on the other hand, is usually produced by the mammalian ovary, corpus luteum, or placenta and may be conjugated and has the widest range of physiological functions [17, 18]. In addition, estrogen is also known to be produced by both maternal and foetal adrenal glands during pregnancy [19, 20]. Some of its actions during pregnancy include, but are not limited to, stimulation of the growth of mammary gland ducts and secretory activities of the oviduct and uterus to enhance foetal survival, regulation of gonadotropin secretion, relaxation of pelvic structures, softening of the pubic symphysis, and general enlargement of the perineal area [21].

The actions of progesterone and estrogens are mediated by their respective nuclear receptors [22]. The regulation of progesterone receptor (PR) genes in the uterine tissue is critical for the response of the organ to progesterone and thus the maintenance of uterine quiescence during gestation. Hence the mechanism regulating progesterone secretion and PR expressions are important in the understanding of uterine physiology during pregnancy. One conserved function of steroid hormone receptors is that they autoregulate the expression of their own genes [23]. In general, hormone receptors are regulated both by their own ligand (homologous regulation) and by other regulatory molecules (heterologous regulation). Endogenous glucocorticoids are known to be involved in the heterologous upregulation of several hormone receptors [24]. The synthetic glucocorticoid, dexamethasone, may have similar role during pregnancy. However, there may be some elements of interspecies and/or breed difference in response to dexamethasone therapy amongst different species, hence the need for further clarification on the effects of dexamethasone on species or breed specific responses in Yankasa sheep. The objective of this study was to assess the influence of dexamethasone on progesterone and estrogen concentrations and uterine progesterone receptor localization in pregnant Yankasa sheep.

## 2. Materials and Methods

### 2.1. Ethical Consideration

All procedures involving the use of animals were reviewed and approved by the Faculty Post-Graduate Committee of the Faculty of Veterinary Medicine, University of Maiduguri, and cleared by School of Post-Graduate Studies, University of Maiduguri, and carried out in accordance with the ethical standards concerning animal welfare as spelt out in the Consensus Guidelines on Animal Ethics and Welfare for Veterinary Journals (International Association of Veterinary Editors, Geneva, Switzerland, 2010).

We followed some aspects of the methods of Yahi et al. [25, 26] in our methodology.

### 2.2. Animals and Management

Twenty healthy adult Yankasa sheep comprising 18 ewes and 2 rams were used for this study. The animals were purchased from main livestock market and private farms in Maiduguri Metropolis. The ages of the ewes ranged between 2 and 3 years, while the rams were 2.5 and 3 years. The ewes weighed between 30 and 38 kg and the rams weighed 35 and 40 kg, respectively. The animals were housed in the University of Maiduguri Livestock Farm and managed intensively. Before the commencement of the experiment, the health status of the animals was evaluated clinically and they were treated prophylactically with oxytetracycline LA (Introxin-200®, Interchemie, Venray, Holland) at 20 mg/kg body weight and ivermectin (Paramectin®, Pharma Swede, Egypt) at 200 *μ*g/kg body weight and were allowed to acclimatize for six weeks. Their feed rations consisted of wheat offals, beans husks, and hay from groundnut leaves. Salt licks and water were provided ad libitum. The rams were kept separate from the ewes until the time of service. Throughout the period of the experiment, these animals were maintained under good conditions with body condition score (BCS) of 3.0 and 3.5 in the 1 to 5 scale.

### 2.3. Estrus Synchronization

The animals were synchronized at the end of the acclimatization period using cloprostenol (Estrumate®, Schering Trough Animal, Germany) given intramuscularly at 250 *μ*g/kg, 11-day interval as reported previously [27]. They were teased with apronned males daily and all the females that came into estrus after the second treatment were allowed to be served naturally by the males. Day of service was recorded and considered as day 0 of the gestation. Pregnancies were confirmed by failure to return to estrus and by ultrasonographic examination using Draminski Ultrasound Pregnancy Detector (UPD-PD032013EX-1.2, Draminsky Agricultural Engineering Co. Inc., Owocowa-Olsztyn, Poland). The animals were then randomly separated into 2 groups of 9 each: dexamethasone treated pregnant sheep (DTS) as treatment group and nondexamethasone treated pregnant sheep (NDS) as control.

### 2.4. Treatments

Animals in the dexamethasone treated group were treated with dexamethasone (Dexaphan®, Pharma Pharmaceuticals, Swede, Egypt) injection given intramuscularly at 0.25 mg/kg body weight on days 1, 3, and 5 during the first trimester; days 51, 53, and 55 in the second trimester; and days 101, 103, and 105 in the third trimester. The animals were observed for possible clinical changes throughout the period of the gestation.

### 2.5. Blood Sample Collection and Analysis

Five ml of blood sample was collected with minimal excitement on day 0 and thereafter on biweekly basis from each animal through the jugular vein and transferred into sterile sample bottles without anticoagulant. The blood was allowed to clot and centrifuged at 3000 rpm for 5 minutes. The serum was harvested and stored at −20°C until assayed for progesterone and estrogen using standard sheep ELISA kits (BlueGene, BioTech Inc., Shanghai, China). The levels of serum progesterone and estrogen were determined through the microtitration plate enzyme-linked immunosorbent assay (ELISA) method according to manufacturer's instruction. For progesterone assay, the minimal detectable concentration was 0.1 ng/ml and the intra- and interassay coefficient of variations for progesterone were 4.5% and 10.8%, respectively. For estrogen, the minimal detectable concentration was 0.62 pg/ml. Intra- and interassay coefficient of variations were 7.1% and 11%, respectively.

### 2.6. Immunohistochemistry

Uterine biopsies were harvested through caesarean section using three (3) pregnant ewes randomly selected from each group at days 28 (first trimester) and 78 (second trimester) of gestation. Three (3) ewes from each group were also allowed to reach full term for normal delivery. The uterine specimens for the analysis of progesterone receptor localization were fixed in 10% neutral buffered formalin for 24 h followed by dehydration in ascending grades of ethanol, cleared in xylene, and embedded in paraffin. Immunohistochemistry was carried out on the paraffin-embedded sections of the uterine specimens. All staining was carried out according to the manufacturer's instructions for paraffin section and used in line with standard protocols as described previously [28]. Fixed tissue molds were cut into sections of 5 *μ*m thick by microtome machine and fixed onto poly-lysine coated precleaned immunohistochemistry tissue slides (1′ × 3′ × 10 mm), deparaffinized in xylene, and rehydrated in a graded ethanol series (100%, 75%, and 50%). Inactivation protocol was used to block endogenous biotin and peroxidases according to the manufacturers' instructions. The actions of tissue-specific endogenous peroxidases were inhibited by incubating sections in prediluted 1% hydrogen peroxide (RE7101) obtained from Novocastra™ kit (Peroxidase Detection System; product number: RE 7110-K, Novocastra Laboratories Ltd., UK) for 3–5 minutes. After washing 2 times (5 min each) in Tris buffer solution (TBS), pH 7.6 (0.5 M Tris HCl and 0.15 M NaCl), sections were placed in 10 mM citrate buffer (pH 6.0) in a microwave oven for 10 minutes followed by cooling to room temperature. Following cooling period, sections were again rinsed twice in TBS, for 3 min. Protein blocking was achieved by incubating with blocking solution (RE 7102) obtained from the Novocastra kit for 10 minutes. After blocking, the mouse monoclonal primary antibodies for progesterone receptor (PR-AT 4.14-Ab2764, Abcam®, UK) with 1 : 60 dilution were added to each slide and were allowed to incubate overnight in a humidified chamber at 4°C.

Afterwards, sections were again gently rinsed in TBS 3 times for 10 minutes each. The sections were then incubated for 30 minutes, at room temperature, in 1 : 300 dilution of biotinylated polyclonal rabbit-anti-mouse secondary antibody (Dako Hamburg, Germany), This was also followed by incubation with preformed avidin-biotin-peroxidase complex (TA-060-PBQ, Ultra Vision-Thermo Fisher scientific Co. Inc., Waltham, USA) and diaminobenzidine (DAB) chromogen (RE 7105) in DAB substrate buffer, Novocastra kit (RE 7106), for 5 minutes each, at room temperature. The sections were then washed in running water for 3–5 minutes and then submerged for 40 seconds in hematoxylin to counterstain.

Following hematoxylin staining, slides were dehydrated in graded alcohol followed by two rounds of xylene and then mounted in DPX Mountant (Sigma, Munich, Germany). Sections of pregnant rat uterus were used as positive control while the negative control was obtained by replacing the primary antibody with TBS on an adjacent section for every treated section.

The processed slides were viewed under light microscope using the Multiple Headed Microscope (DESC-LN-0100-MG001, Vamed Engineering, UK), for the progesterone receptors staining. Photomicrographs were taken using the Canon IXUS Camera, China, with pixel: 16.5. The staining intensity and the positive cells were evaluated semiquantitatively and scored visually by two observers as described by Diest et al. [29] and modified by Vermeirsch et al. [30]. This involved systematic and random counting of sampled fields of vision, including luminal, glandular, stromal, and myometrial epithelia, and the percentages of the positive cells were calculated. Ten (10) random fields in three technical biological replicates were counted to reach a minimum count of 200. At 100x magnification in each field, the numbers of cell with the different staining intensities were counted using set up numbers from 0 to +4. Negative, weak, moderate, strong, and very strong positive staining were evaluated and scored visually on a scale of 0 to 4, respectively, as previously described [29, 30]. The intensity score (“histo-” score, H-score) was generated using the formula: H-score = ∑(1 + *i*)*p*_*i*_, where *i* is the staining intensity numbers and *p*_*i*_ is the percentage of cells showing that intensity [31]. Means ± SD for the score were generated and *t*-test was applied for statistical significance.

### 2.7. Statistical Analyses

Data collected were expressed as means** ±** standard deviations (SD) and managed in MS Excel worksheet to generate data charts. The significant differences between the dexamethasone treated and nondexamethasone treated groups were compared using Student's *t*-test. Significant differences were considered at *p* < 0.05. Computer statistical software package, GraphPad InStat®, [32] was used for the analysis.

## 3. Results

**Figure 1:**
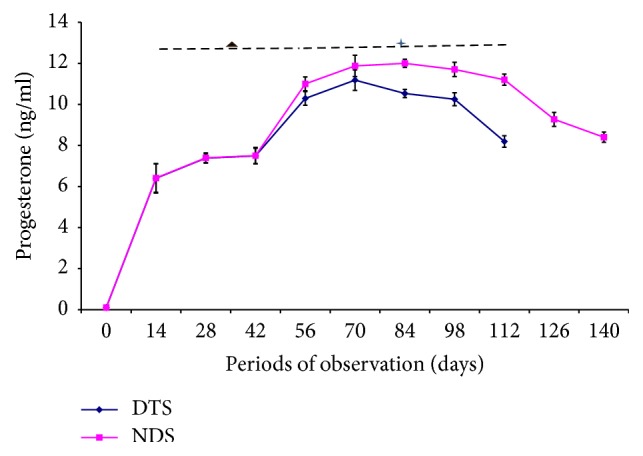
Effects of dexamethasone on progesterone concentration in dexamethasone treated pregnant sheep (DTS) and nondexamethasone treated pregnant sheep (NDS). Four-pointed star: significantly lower (*p* < 0.05) and triangle: insignificant (*p* > 0.05) in DTS compared to NDS.

**Figure 2:**
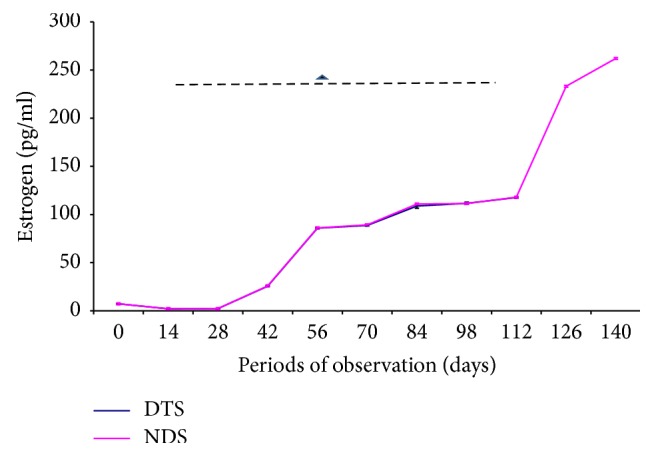
Effects of dexamethasone on estrogen concentration in dexamethasone treated pregnant sheep (DTS) and nondexamethasone treated pregnant sheep (NDS). Triangle: insignificant (*p* > 0.05) in DTS compared to NDS.

The changes in serum estrogen and progesterone concentrations in pregnant Yankasa sheep following the administration of dexamethasone are presented in Figures 1 and 2. While progesterone concentration increased progressively with advancing pregnancy in both the treated and control groups up to the second trimester, there were significant (*p* < 0.05) decreases at time-points in serum progesterone concentrations in dexamethasone treated sheep compared to the control (Figure 1). This decrease was observed at the beginning of the second trimester (day 56) and continued up to day 112 of gestation compared to the control for the same period. However, there was no significant (*p* > 0.05) variation in estrogen concentrations between dexamethasone treated group and the respective control group (Figure 2).

**Figure 3:**
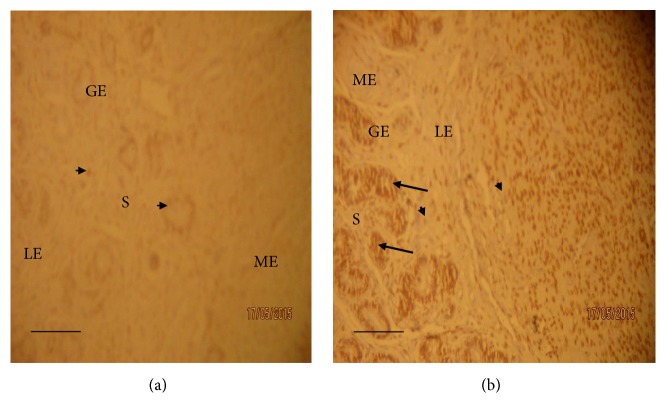
Progesterone receptor staining of Yankasa sheep uteri during first trimester (day 28 of gestation). Arrow heads indicate moderate positive progesterone receptor (PR) staining (2+) and eosinophilic cells, while arrow indicates strong positive staining (3+) for progesterone receptors (PR) and endometrial lymphocytes. Figure 3(a) (control sheep): progesterone receptor staining showed moderate positive staining (2+) in all parts of the uterus (IHC ×100). Figure 3(b) (dexamethasone treated sheep): progesterone receptor staining indicated strong positive staining (3+) in the glandular epithelia cells (GE), moderate positive staining (2+) in the stromal (S) and luminal cells (LE) (IHC ×100), and traced staining (1+) or moderate staining (2+) in the myometrial cells (ME). Scale bars represent 50 *μ*m.

**Figure 4:**
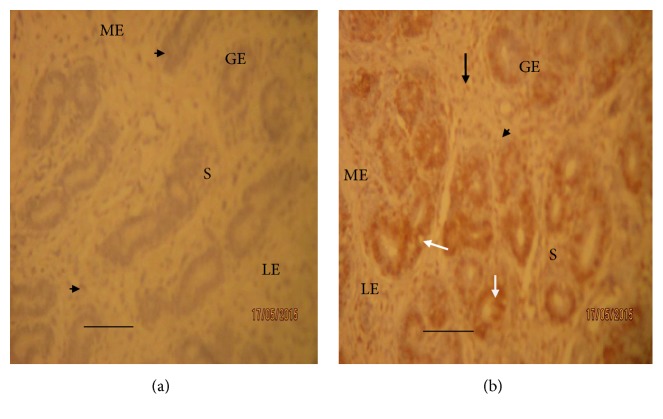
Progesterone receptor staining of Yankasa sheep uteri at day 78 of gestation. Arrowheads indicate moderate positive progesterone receptor (PR) staining (2+) and eosinophilic cells, while black arrow indicates strong positive progesterone receptor (PR) staining (3+). White arrows indicate very strong positive staining (4+) for progesterone receptors (PR) and endometrial lymphocytes.  Figure 4(a) (control sheep): progesterone receptor staining showed moderate positive staining (2+) in all parts of the uterus (IHC ×100).  Figure 4(b) (dexamethasone treated sheep): progesterone receptor staining pattern showed moderate positive staining (2+) in the luminal epithelia (LE) and myometrial cells (ME), strong positive staining (3+) in the stromal epithelial (S), and very strong positive staining (4+) in the glandular epithelia cells (GE) (IHC ×100). Scale bars represent 50 *μ*m.

On the other hand, the immunohistochemical evaluation of the gravid uteri showed that progesterone receptor (PR) was more intensely upregulated in the dexamethasone treated Yankasa ewes uteri compared to control group (Figures 3 and 4). The immunoreactivity for progesterone receptors was localized in the nuclei of the positive cells of both treated and control groups as indicated by arrows and arrow heads in the plates (Figures 3 and 4). There were abundant progesterone receptors for nuclear staining of both the glandular and luminal cells and the majority of stromal cells and myometrial cells. However, intense progesterone receptor concentrations were mostly expressed in the glandular epithelia of the endometrial glands compared to the luminal, stromal, and myometrial cells. Hence in glandular epithelia, staining intensity for uterine progesterone receptors was observed to be strongly positive (3+) in dexamethasone treated sheep, moderate positive staining (2+) in the stromal and luminal cells, and traced (1+) in the myometrial cells at day 28 of gestation (Figure 3(b) and Table 1). On the other hand, the staining intensity for progesterone receptor during first semester in the control group of Yankasa sheep remained moderate (2+) in all parts of the uteri (Figure 3(a) and Table 1).

During second trimester (day 78), the staining characteristics in the control group uteri did not change but remained at the level of moderate (2+) staining in all parts of the uteri. However, in the dexamethasone treated group, the staining intensity increased from moderate positive (2+) to strong positive (3+) in the luminal and stromal cells and from strong (3+) to very strong positive (4+) intensity in the glandular epithelial cells, while in the myometrial cells, the intensity increased from traced staining (1+) to moderate positive (2+) by day 78, during second trimester (Figures 4(a) and 4(b) and Table 1). Similarly, during first trimester, the intensity scores were significantly (*p* < 0.05) higher only in the glandular epithelia of the dexamethasone treated group compared to that of control. On the other hand, the intensity scores increased and became significantly (*p* < 0.05) higher in all sections of the endometrial tissues in the dexamethasone treated group compared to control (Table 1).

Three ewes from the dexamethasone treated group had abortion on day 120 of gestation. The mean aborted foetal weight was 974 ± 0.50 g, while the mean aborted placental weight was 365 ± 0.23 g. Three ewes from the control group had normal parturition: one on day 147 and two on day 148 of gestation.

## 4. Discussion

In this study, dexamethasone treatment caused abortion in sheep during advanced pregnancy which could be associated with decreasing progesterone concentration in systemic circulation. McDonald et al. [33] observed similar phenomenon in rats, but the mechanism of decrease was not very clear. However, from the present study, the significant decrease in progesterone concentration in the dexamethasone treated sheep may be in part, due to the adverse effects of dexamethasone on placenta. Dexamethasone has previously been reported to have adverse effects on placenta like decreased placental weights and efficiency [2, 5–11].

The literature has shown that progesterone is mainly produced by corpora lutea (CL) and placenta during pregnancy [13, 15]. However, in sheep progesterone is mainly produced by placenta with little contribution from the corpus luteum especially during advanced pregnancy [34–38]. Therefore the maintenance of normal progesterone concentration and pregnancy in sheep is placenta-dependent. In previous study, decreased placental weight was observed in the dexamethasone treated sheep which could have compromised placental efficiency and led to lowered progesterone production and secretion [24].

During pregnancy uterine quiescence is maintained by elevated levels of circulating progesterone acting through its receptor (PR), whereas decreasing progesterone concentration or activity promotes parturition or abortion [39, 40]. The low progesterone concentration could be the possible cause of abortion in dexamethasone treated sheep in this study. Progesterone inhibits prostaglandin synthesis and activity in pregnant subjects [41] and consequently decreases myometrial contractility. This inhibition is mediated by a number of pathways that include blocking prostaglandin action, decreasing prostaglandin synthesis, and increasing its rate of inactivation [41]. A fall in progesterone concentration during pregnancy is associated with increased prostaglandin synthetase activity and prostaglandin F_2_*α* production that can predispose to abortion [42].

The decreased progesterone concentration observed in this study is similar to that reported by Ahmadabad et al. [43] in pregnant mice treated with dexamethasone, but Gale [44] and Ohrlander et al. [45] who worked with dexamethasone to induce foetal lung maturation in human did not observe any alteration in the plasma concentrations of progesterone. In the present study, dexamethasone treatment did not alter circulating estrogen level during pregnancy in the Yankasa sheep although Ohrlander et al. [45] previously reported suppression of estrogen production in pregnant women by dexamethasone. These differences may be due to differences in source progesterone secretion during pregnancy. The primary source of progesterone during pregnancy in human is the corpus luteum (CL). The placenta does not contribute substantially to progesterone production during pregnancy until mid-gestation. Therefore the negative influence of dexamethasone on placenta as reported in literature may not have significant effects on progesterone and estrogen production. In humans and other primates in particular, maintenance of the corpus luteum itself is favored by the hormone, Human Chorionic Gonadotropin (hCG). This hormone has luteinizing hormone- (LH-) like activity that protects the corpus luteum from regression and stimulates its production of progesterone. If conception occurs, the corpus luteum is maintained and grows and secretes increasing amounts of progesterone. Hence in humans, the corpus luteum continues to produce progesterone until around mid-gestation when placenta begins substantial contribution to progesterone production and release [46].

The intensity score (“histo-” score, H-score) is a method of assessing semiquantitatively the extent of nuclear immunoreactivity, applicable to steroid receptors [31]. The increase in the intensity scores and hence the upregulation of the PR in dexamethasone treated Yankasa sheep uteri during pregnancy could be one of the beneficial effects of dexamethasone. The upregulation is probably, in part, a result of compensatory mechanism response in order to increase progesterone sensitivity and enhance chances of interaction between the PR and the hormone as the data indicated that dexamethasone significantly decreased progesterone levels (Figure 1). Receptors of circulating hormones are typically upregulated when the concentration of the hormones are decreased in order to increase the sensitivity of the receptors and the chances of their interactions with the hormones [24].

Glucocorticoids are known to be involved in the heterologous upregulation of several hormone receptors [24]. Dexamethasone, being a synthetic glucocorticoid, might have played a similar role in the upregulation of PR in Yankasa sheep in this study. This compensatory mechanism may possibly explain the intense rise in progesterone receptors observed by mid-gestation in the dexamethasone treated sheep compared to control in this study and may probably be channeled through the regulation of receptor mRNA levels by influencing increase in PR mRNA levels and gene transcription as earlier reported in rats [47] and humans [48]. Thus, dexamethasone probably stimulated transcriptional activity of PR and increased total PR expression in the gravid uteri.

## 5. Conclusion

Dexamethasone decreased progesterone concentrations and caused abortion in Yankasa sheep but no aberrant effect on estrogen concentrations was observed. Progesterone receptors (PR) were strongly upregulated in the second trimester compared to the first trimester. The abortions observed in the second trimester were caused by decreased progesterone concentrations probably as a consequence of adverse effects of dexamethasone on placenta since sheep progesterone production and secretion during pregnancy are placenta-dependent. The PR upregulation may be a compensatory mechanism to increase their sensitivity and the chances of their interactions with progesterone in the phase of decreasing progesterone concentration as a direct consequence of the dexamethasone. The intense progesterone receptor concentrations observed were mostly expressed in the glandular epithelia of the endometrial glands compared to the stromal and myometrial cells. Repeated dexamethasone therapy should therefore not be used in advanced pregnancy in sheep and other placenta-dependent progesterone secreting pregnant subjects.

## 6. Recommendation

Previous studies showed that fat-supplemented diets increased serum progesterone concentration in some species of animals, like cows and sheep [40, 49]. Therefore, it is recommended that fat supplemental diet should be provided in animals under dexamethasone therapy to augment for the progesterone decrease.

## Figures and Tables

**Table 1:** Effects of dexamethasone on uterine progesterone receptor localization and intensity scores in pregnant Yankasa sheep during first and second trimesters.

Endometrium	Group^*∗*^	First trimester (day 28)		Second trimester (day 78)	
		Staining intensity	Intensity score	Staining intensity	Intensity score
Luminal	NDS	2+	61.18 ± 0.22	2+	60.45 ± 0.56
	DTS	2+	61.24 ± 0.20	3+	87.50 ± 1.20^a, b^
Glandular	NDS	2+	60.50 ± 0.63	2+	61.19 ± 0.30
	DTS	3+	89.11 ± 0.94^a^	4+	134.10 ± 1.08^a, b^
Stromal	NDS	2+	62.37 ± 0.43	2+	61.56 ± 0.51
	DTS	2+	62.41 ± 0.30	3+	86.49 ± 0.45^a, b^
Myometrium	NDS	1+	51.58 ± 0.55	1+	52.32 ± 0.37
	DTS	1+	52.24 ± 0.22	2+	60.80 ± 0.35^a, b^

Total	NDS	—	234.71 ± 1.26	—	235.40 ± 1.10
	DTS	—	267.14 ± 1.92^a^	—	366.28 ± 1.70^a, b^

NDS: (control) nondexamethasone treated pregnant sheep; DTS: dexamethasone treated pregnant sheep; ^*∗*^*N* = 3 for each group.  ^a^Significant (*p* < 0.05) increase compared to respective control group. ^b^Significant (*p* < 0.05) increase compared to preceding gestational stage.
